# Compensation profiles among private sector employees in Sweden: Differences in work-related and health-related outcomes

**DOI:** 10.3389/fpsyg.2023.949711

**Published:** 2023-02-28

**Authors:** Alexander Nordgren Selar, Marylène Gagné, Johnny Hellgren, Helena Falkenberg, Magnus Sverke

**Affiliations:** ^1^Department of Psychology, Division of Work and Organizational Psychology, Stockholm University, Stockholm, Sweden; ^2^Future of Work Institute, Faculty of Business and Law, Curtin University, Perth, WA, Australia

**Keywords:** compensation, health, organizational justice, leadership, pay, pay dispersion, job performance, turnover

## Abstract

How experiences and perceptions of pay and pay setting relate to employees’ job performance, willingness to remain in the organization, and health has been the subject of much debate. Previous research has typically used a variable-centered approach to investigate associations between different pay-related factors and such outcomes. In contrast, we used latent profile analysis to explore combinations of compensation characteristics (pay level, perceived horizontal pay dispersion, and procedural quality, i.e., transactional leadership and procedural pay-setting justice), combining relevant theories on the subject. Based on a nationally representative sample of private sector employees in Sweden (*N* = 1,146), our study identified six compensation profiles. Our key findings show, first, that higher levels of pay were generally associated with better performance, lower turnover intention, better self-rated health, and lower work-related exhaustion, especially when combined with perceptions of high procedural quality. Second, in terms of perceived horizontal pay dispersion, the results indicate that pay compression may be associated with beneficial outcomes, particularly when combined with high procedural quality. Third, procedural quality was generally associated with favorable work-related and health-related outcomes, although such positive effects may be contingent upon pay level and perceived horizontal pay dispersion. In conclusion, while pay level, perceptions of horizontal pay dispersion, and procedural quality may all matter for employee outcomes, it is important to consider their combinations.

## 1. Introduction

Pay setting is often used as a managerial tool, because it is believed to be effective in attracting, motivating, and retaining staff (Gerhart and Fang, 2015). A wide variety of compensation and incentive systems are in use in today’s workplaces (Cameron, 2022). This includes compensation systems where, for instance, seniority or the general degree of responsibility demanded for the job are the determinants of pay (Pfeffer, 1997, 1998; Bloom, 1999) as well as performance-based systems in which pay is partly based on individual performance, including the degree to which individuals reach the goals of their jobs and fulfill their responsibilities for certain activities (Maaniemi, 2013). In this research, we investigate which pay-setting characteristics (and their combinations) that may be central to employee outcomes, addressing the question of whether it is indeed an effective managerial tool to motivate performance and retain employees. In addition, we investigate what effects these characteristics may have on employee health.

The way pay setting should be carried out in order to contribute to employee performance and retention is a major issue for organizations and is debated among researchers (e.g., Shaw and Gupta, 2015; Lazear, 2018; Gagné and Forest, 2020). In recent years, some research attention has also been given to the effect of organizations’ pay setting on employee health (Cadsby et al., 2016; Allan et al., 2020; Dahl and Pierce, 2020). One question has concerned whether compensation systems should be compressed (i.e., fixed pay agreements, where colleagues with similar jobs are equally paid) or dispersed (i.e., incentivized pay agreements, where pay is partly based on individual performance) in order to stimulate performance, retention, and health (Pfeffer and Langton, 1993; Shaw, 2014). Another question has been how experiences of justice in the pay-setting process relate to these kinds of outcomes (Olafsen et al., 2015; Malmrud et al., 2020). A third question concerns leadership style, where it has been argued that managers’ ability to explain the bases of pay decisions is central for pay to affect employee performance and health (*cf.* Rowold and Schlotz, 2009; Han et al., 2015). Fourth, other research contends that what really matters is the pay level and that higher levels of pay are related to better performance and lower turnover (*cf.* Kuvaas et al., 2016; Thibault-Landry et al., 2017) as well as better health (*cf.* Marmot et al., 1991). Notably, in Sweden (where this study was conducted), this discussion has increased in strength in recent decades (Hellgren et al., 2017; Ulfsdotter Eriksson et al., 2021) in connection with a gradual increase in performance-based pay systems (especially among public sector employees, but also in the private sector; Swedish National Mediation Office, 2017).

Both international (Shaw, 2014; Gagné et al., 2023) and Swedish (Lundh, 2010; Hellgren et al., 2017) perspectives on the matter have, however, acknowledged that it is most likely a combination of different pay-related factors that contributes to employees’ performance, willingness to remain with their organization, and health. This paper uses a person-centered approach (e.g., Bergman and Magnusson, 1997) in order to study different combinations of compensation characteristics. Such combinations are difficult to detect with a variable-centered approach, since interactions between several variables become difficult to interpret (Howard et al., 2016). In contrast to investigating associations between various predictors (e.g., pay-related factors) and an outcome, a person-oriented approach focuses on the effects of different combinations of characteristics and perceptions regarding pay setting.

The overall aim of the present study was to increase the general understanding of how different combinations of employees’ perceptions of pay and pay setting may contribute to job performance, retention, and health-related outcomes. More specifically, the first aim was to identify groups of individuals with similar pay levels, perceptions of pay differences among coworkers (i.e., perceived horizontal pay dispersion), perceptions of reward-emphasizing leadership (i.e., transactional leadership), and perceptions of procedural fairness (i.e., procedural pay-setting justice), here labeled compensation profiles. Our second aim was to explore differences between these compensation profiles in terms of work-related outcomes (task performance and turnover intention) and health-related outcomes (self-rated health and work-related exhaustion). To increase our understanding of such differences, the third aim was to describe how the compensation profiles differ in demographic background variables (age, education level, sex, managerial status, and employment status) and psychosocial work environment factors (job demands, job control, and social support).

This study contributes to the literature in several ways. First, the person-centered approach allows for investigating combinations of several variables, which is important in examining the contribution of different features of a pay system simultaneously. This would be very difficult to achieve using a variable-centered approach as it would mean analyzing interactions between several variables. To our knowledge, a person-centered approach has not been used in the pay-setting context before. Second, by using a large and nationally representative sample of private sector employees in Sweden, not only does the study contribute to the understanding of general industry tendencies, but it allows to obtain variability in pay characteristics necessary to understand how these combinations may influence employee outcomes. Third, by including a wide range of outcomes, the study contributes to the growing body of research considering that employee pay and perceptions of pay setting do not only concern work-related outcomes such as task performance and turnover intention but could also concern employee health such as self-rated health and exhaustion (Parker et al., 2019; Dahl and Pierce, 2020).

### 1.1. Different perspectives on pay and pay setting

Several compensation characteristics (e.g., pay differences and pay procedures) are likely to influence job performance, turnover intention, and health. The theoretical perspectives used to explain such outcomes use noticeably different assumptions about work motivation, which has given rise to widely differing predictions about how various compensation characteristics relate to work-related and health-related outcomes.

One perspective concerns expectancy theory (Vroom, 1964), which many contemporary perspectives have relied on to predict that rewards based on meeting performance criteria will enhance employee motivation and, further, that opportunities to strive for economic rewards will motivate employees to want to stay in their jobs (Shaw, 2014). Relatedly, tournament theory (Lazear and Rosen, 1981; Lazear 1995, 2018) proposes that pay differentials based on performance can motivate workers to want to be “at the top,” inspiring talented employees to continue to achieve, and motivating those who underperform to increase their efforts to want to be as good as their more-rewarded peers (i.e., a motivational effect). If tournaments over pay do not increase the motivation of underperformers, organizations can choose to reward them less than others, and by doing so, signal that they are underperforming, which might lead them to start searching for a job in another organization (e.g., Shaw, 2014; Lazear, 2018). In line with expectancy and tournament theories, the role of managers is to make the performance–reward connection salient for the employees (Han et al., 2015).

A second perspective is rooted in justice theories (e.g., Rawls, 1971; Leventhal, 1980; Greenberg, 1987; Colquitt, 2001) which suggest that the methods used to determine compensation also matter, in that they need to be perceived as procedurally fair (e.g., Olafsen et al., 2015; Malmrud et al., 2020). Justice, in this context, depends on the extent to which pay decisions are consistent, objectively made, and based on correct information (Stråberg, 2010). Equity theory complements this perspective by proposing that people compare what they have obtained to the effort that they have put in and to what others engaged in similar work have obtained, and thus expect to be rewarded accordingly (Adams, 1965). The fairness perspective thus suggests, in line with meta-analytic results on the role of organizational procedural justice (Colquitt et al., 2001, 2013), that if pay setting in organizations is perceived as fair, it has a better chance of resulting in positive work-related and health-related outcomes (Malmrud et al., 2020).

A third perspective is represented by self-determination theory (SDT; Deci and Ryan, 1985), which argues that organizations should avoid making compensation salient in organizations in order to keep employees focused on autonomous sources of motivation (e.g., meaning and/or personal importance) rather than on extrinsically controlling sources (e.g., rewards, ego-boosts, or avoiding sanctions). According to SDT research, autonomous work motivation has a strong positive impact on employee performance, retention, and health. Extrinsically controlled work motivation, on the other hand, often fails in these regards (Cerasoli et al., 2014; Gagné et al., 2015; Van den Broeck et al., 2021). Based on this, SDT suggests that rather than increasing the salience of economic rewards (which may result in extrinsically controlled work motivation), organizations should decrease the salience of economic rewards and concentrate on actions that encourage autonomous work motivation. To encourage autonomous work motivation, organizations should thus focus on strategic plans and work designs that aim at making work more meaningful and engaging (*cf.* Deci et al., 1999; Gagné and Deci, 2005; Gagné, 2018; Gagné and Forest, 2020). Stress theories add to SDT a special health perspective on compensation characteristics (e.g., Ganster et al., 2011) by emphasizing that performance–reward connections in organizations may increase performance pressure (which is already very high in a wide range of today’s industries; Pfeffer, 2018), thereby adding additional stress (Parker et al., 2019).

## 2. Compensation system characteristics and their associations with outcomes

Previous research has highlighted that a number of compensation characteristics are of particular theoretical and practical importance for employee outcomes such as performance, retention, and health (e.g., Shaw, 2014; Han et al., 2015; Olafsen et al., 2015; *cf.* Parker et al., 2019). This section addresses four such compensation characteristics, namely, pay level, perceived horizontal pay dispersion, transactional leadership, and procedural pay-setting justice.

### 2.1. Salient compensation characteristics

#### 2.1.1. Pay level

The level of pay represents what employees receive in exchange for their work according to their contract with the organization (Lawler, 1971). Contracted pay serves to secure the fulfillment of basic survival needs (e.g., food, shelter, and basic safety); those whose salaries more than cover such needs may then be expected to be able to devote more of their energy to the work itself (Howell et al., 2013). Furthermore, offering sufficient levels of pay that compare well to pay levels in competing organizations might curb the competition of labor, while specifically setting pay at higher levels than competing organizations might also help organizations to attract and retain skilled employees (He et al., 2016). A high pay level can also signal that the employee is highly valued, making them feel more competent and increasing their desire to “belong” in the organization (Thibault-Landry et al., 2017). The level of pay can also exert a degree of impetus for the employee’s effort and motivation (Locke et al., 1980).

Previous research indicates that receiving high pay—or at least a satisfying level of pay—can positively impact employee performance (*cf.* Kuvaas et al., 2016) and health (Ettner, 1996), and high levels of pay have been shown to improve self-esteem, which, in turn, might decrease turnover intention (Gardner et al., 2004). However, there could be ceiling effects for pay level (i.e., that the importance of pay drops after a certain, sufficient, limit; Jebb et al., 2018) and marginal utility of money effects (i.e., that the importance of money lessens as consumption demands are met; Sieweke et al., 2017).

#### 2.1.2. Perceived horizontal pay dispersion

Pay dispersion refers to the level of pay-related inequality, for example, in an entire organization or between specific groups of employees (Bloom, 1999). It can be operationalized as the actual differences in pay between employees (actual pay dispersion) or as perceived differences in pay by employees (perceived pay dispersion; *cf.* Card et al., 2012). Pay dispersion may be conceptually divided into horizontal pay dispersion (i.e., pay differences among those on the same level in an organization, such as coworkers) and vertical pay dispersion (i.e., pay differences between employees on different levels in the organization, such as between blue-collar workers and the top management; Shaw, 2014). In this study, we focus on perceived horizontal pay dispersion because this is the type of pay dispersion that typically develops in organizations using performance-based pay systems (e.g., through its annual performance-based pay raises that increase differences in employee pay). Such differences can also occur in organizations, for example, whose employee pay is influenced by employment tenure (Shaw, 2014). In addition, we focus on perceived pay dispersion because theory (e.g., tournament theory) predicts that the expected positive effects on job performance and retention occur when the pay differences are apparent to employees (Lazear, 2018). Perceived pay dispersion may or may not be in line with the actual pay dispersion levels in organizations, and the level perceived depends on how much information employees have about others’ pay (i.e., pay transparency; Card et al., 2012).

In previous research, actual high pay dispersion levels have been shown to relate to certain areas of higher job performance, such as regarding organizational productivity (e.g., Lallemand et al., 2009), sales growth (Shaw, 2015), and higher competitive performance (Becker and Huselid, 1992). However, actual high pay dispersion has also been shown to relate to lower individual and team performance (Bloom, 1999; Bucciol et al., 2014), lower research productivity (Pfeffer and Langton, 1993), and lower team innovation (Yanadori and Cui, 2013). Furthermore, research has found that actual high horizontal pay dispersion is related to increased turnover across many organizational settings and many countries; it was associated with increased turnover intention not only at all pay levels when the pay setting was not based on performance but also particularly among poor performers when the pay setting was based on performance (Shaw, 2014). Concerning perceptions of horizontal pay dispersion, previous research has found that when employees were aware of the pay differences among them and their coworkers (because they were disclosed), those finding themselves worse off had lower job satisfaction and increased turnover intention; those who were pay-favored employees, however, were unaffected by the disclosure of their relative favoritism (Card et al., 2012).

There has been little research on health-related outcomes in relation to pay dispersion. However, research has indicated that status hierarchies in organizations (which could be reflected through a dispersed pay structure) might lower well-being (Christie and Barling, 2010), and increase risks for ill-health (*cf.* Marmot et al., 1991). In addition, the increased use of pay systems that generate performance-based pay differences among non-managerial employees in private companies and public organizations (Pfeffer, 1997) has been associated with increased stress among workers, according to a study from Denmark (Dahl and Pierce, 2020).

#### 2.1.3. Transactional leadership

One option for managers to influence the amount of effort employees put into their work is to monitor employees’ work behaviors and communicate to them how their behaviors could affect how they are rewarded in the future (i.e., adopting a transactional leadership style; Yukl, 1999; Rowold and Schlotz, 2009; Han et al., 2015). This may be achieved by emphasizing to employees what they must do in order to receive rewards, providing feedback on whether they act accordingly, and rewarding those who do (Han et al., 2015; Young et al., 2021). Furthermore, it has been theorized that by making the performance–reward connection salient—through managers having discussions with their employees about the rewards available and helping them to prioritize the associated work tasks—leaders can create role clarity and certainty, and thereby facilitate stress reduction among their employees (*cf.* Rowold and Schlotz, 2009). Overall, transactional leadership can thus strengthen an organization’s reward systems, thereby increasing the instrumentality and valance of the pay system, which is advocated by perspectives (e.g., Han et al., 2015) inspired by expectancy theory (Vroom, 1964).

It has been rather well established in previous research that the use of transactional leadership is positively associated with work-related (Judge and Piccolo, 2004) and health-related outcomes (see Rowold and Schlotz, 2009), although the use of transformational leadership (i.e., a supportive and inspiring leadership style; Yukl, 1999) has evidenced stronger positive effects on such outcomes (e.g., Judge and Piccolo, 2004; Wang et al., 2011; Zwingmann et al., 2014). However, the role of transactional leadership in organizations’ pay setting is more controversial. On the one hand, some previous research has shown that transactional leadership enhances performance-based pay systems’ ability to drive performance (Han et al., 2015). On the other hand, SDT (Deci and Ryan, 1985) holds that transactional leadership risks robbing employees of their sense of autonomy (Ryan and Deci, 2017). According to SDT, this may provide an explanation for the relatively worse effects of transactional leadership on work outcomes, as compared to transformational leadership, as there is more of a risk that the former will stimulate extrinsically controlled work motivation (*cf.* Eyal and Roth, 2011). Relatedly, previous research has shown that compensation systems characterized by a salient reward-related focus are associated with increased workplace stress (Parker et al., 2019) and lower individual performance quality (Cerasoli et al., 2014). This might be explained by the emphasis on extrinsically controlling sources (e.g., that leaders mainly promote employee motivation by highlighting rewards) lowering employees’ autonomous work motivation (Gagné and Forest, 2020).

#### 2.1.4. Procedural pay-setting justice

Procedural justice regarding employee pay is determined by the quality of the pay-setting procedures as well as by the degree of employee acceptance of the pay-setting results (Stråberg, 2010). It relies on the same type of principles as organizational procedural justice (see Colquitt, 2001), but relates more specifically to how pay is set. It has been argued that pay-dispersed compensation systems—when they are characterized by strong procedural fairness—can drive employee performance (Shaw, 2014). However, only a few studies have examined the role of perceived procedural pay-setting justice in performance appraisal and pay determination. One study has found that performance-based compensation only produced the intended performance-increasing effects when there was a strong procedural justice climate in the organization (Sung et al., 2017). Another study found that high levels of procedural justice in pay determination (regardless of the amount of pay received) were positively related to employees’ autonomous motivation (Olafsen et al., 2015). Procedural justice in pay setting has also been linked to reward satisfaction and, through this, to lower turnover intentions (Tekleab et al., 2005).

##### 2.1.4.1. Procedural quality

Based on this knowledge of expectancy theory (Vroom, 1964), transactional leadership (Han et al., 2015), and procedural pay-setting justice (Shaw, 2014), it may be assumed that leadership and fairness together reflect a kind of quality marker regarding pay setting in organizations. Thus, these perspectives highlight transactional leadership (which at a high level is expected to, e.g., lead to higher reward-related expectations) and procedural justice (which at high levels is assumed to legitimize the wage outcome) as complementary elements in an organization’s compensation system that could positively impact work-related and health-related outcomes among employees.

### 2.2. Identifying profiles

Since analyzing the associations between multiple compensation characteristics is statistically demanding through variable-centered regression techniques—especially because of the difficulty of interpreting interactions that involve more than three variables (Howard et al., 2016)—we used a person-centered approach that allows for identifying groups of individuals with similar pay-setting circumstances [i.e., latent profile analysis (LPA); Gibson, 1959]. Based on individual respondents’ responses to a number of different variables, LPA separates the study population into subgroups such that individuals with similar responses across the measures are placed in the same profiles (Morin et al., 2018). It is then possible to examine how these compensation profiles relate to work-related and health-related outcomes. In terms of practice, this approach may help identify how compensation characteristics should best be combined to create compensation systems that support employees as well as organizational development and sustainability.

## 3. Materials and methods

### 3.1. Sample and procedure

Questionnaires were sent in 2016 to a nationally representative sample of 5,000 individuals, aged 20–65 years, employed in the Swedish private sector. The sample selection and administration of the questionnaires were handled by Statistics Sweden. A pre-notification of the project by post was followed by a letter describing the research project, that participation in the questionnaire was voluntary, and that all data collected would be kept confidential. Out of the 5,000 employees sampled for the survey, 1,252 individuals returned their questionnaires, for a response rate of 25%. Among these, 76 cases were excluded because of extensive missing data on the profile predictor variables (i.e., the compensation characteristics), outcome variables, demographic background variables, and/or psychosocial work environment factors. Another 30 cases were excluded because of extreme values (i.e., multivariate outliers) in the profile predictor variables. As a result, 1,146 cases were included in the final sample.

The mean age was 47 years (SD = 11) and the proportion of women was 29%. The average pay was 33,300 (SD = 9,900, range 16,800–69,500) Swedish crowns per month (1,000 Swedish crowns was approx. $117 or €106 in 2016). A non-response analysis showed that dropout was slightly higher among those who were younger (20–26 years), those with a low level of education (primary or secondary level), and those with below average salaries.

The data collection received ethical approval by the Regional Ethics Committee in Stockholm (ref. no. 2015/1733-31/5).

### 3.2. Measures

Table 1 presents descriptive statistics (means and standard deviations) and the intercorrelations for all study variables, along with the reliability estimates (Cronbach’s alpha) for the multi-item measures.

**Table 1:** Means, standard deviations, reliability coefficients (Cronbach’s alpha coefficients in the diagonal), and bivariate correlations for the study variables.

	1	2	3	4	5	6	7	8	9	10	11	12	13	14	15	16
Compensation characteristics																
1. Monthly pay level (log)	–															
2. Perceived horizontal pay dispersion	0.10*	–														
3. Transactional leadership	0.21*	−0.06	(0.78)													
4. Procedural pay-setting justice	0.23*	−0.18*	0.54*	(0.86)												
Work-related outcomes																
5. Task performance	−0.04	−0.05	0.12*	0.20*	(0.79)											
6. Turnover intention	−0.10*	0.14*	−0.20*	−0.23*	−0.10*	–										
Health-related outcomes																
7. Self-rated health	0.06*	0.01	0.17*	0.19*	0.22*	−0.06*	–									
8. Work-related exhaustion	−0.10*	0.12*	−0.15*	−0.23*	−0.12*	0.25*	−0.25*	–								
Demographic background variables																
9. Age (years)	0.11*	−0.10*	0.00	−0.06*	0.01	−0.14*	−0.06*	−0.09*	–							
10. Education (university)	0.27*	0.09*	0.02	−0.00	−0.03	0.09*	0.03	−0.06*	0.07*	–						
11. Sex (woman)	−0.13*	0.10*	−0.06	−0.05	0.11*	−0.00	0.05	−0.01	−0.05	0.16*	–					
12. Managerial status (manager)	0.30*	0.02	0.23*	0.13*	0.04	−0.04	0.03	0.02	−0.03	0.01	−0.10*	–				
13. Employment status (white collar)	0.56*	0.17*	0.22*	0.13*	−0.02	−0.03	0.08*	−0.07*	−0.01	0.31*	0.07*	0.47*	–			
Psychosocial work environment factors																
14. Job demands	0.08*	0.16*	−0.07*	−0.15*	−0.15*	0.15*	−0.09*	0.53*	−0.05	0.06	−0.02	0.11*	0.07*	(0.74)		
15. Job control	0.32*	−0.03	0.38*	0.42*	0.33*	−0.24*	0.23*	−0.23*	0.01	−0.03	−0.06	0.24*	0.26*	−0.16*	(0.79)	
16. Social support	0.07*	−0.06*	0.49*	0.45*	0.20*	−0.23*	0.19*	−0.21*	−0.09*	−0.02	0.04	0.09*	0.08*	−0.20*	0.41*	(0.83)
Mean	10.37	3.03	2.82	3.24	3.92	1.83	4.08	3.19	47	0.45	0.29	0.21	0.54	3.35	3.74	3.51
Standard deviation	0.27	1.36	1.01	1.02	0.69	1.25	0.81	1.17	11	–	–	–	–	0.98	0.94	0.91

*p < 0.05. – Not applicable.

#### 3.2.1. Compensation characteristics

The present study included measures regarding compensation characteristics (used for the identification of profiles encompassing individuals with similar circumstances and perceptions). Monthly pay level was measured with a single question, “How much (in Swedish crowns) do you usually earn each month through your regular employment (before the tax deduction)?” Following conventional practice in previous income-related research (e.g., Sieweke et al., 2017), we transformed each participant’s pay level amount into a log value using a natural logarithm. Perceived horizontal pay dispersion was measured with one item, “To what extent are there salary differences among employees with similar jobs at your workplace?” with a response scale from 1 (to a very small extent) to 5 (to a very large extent). Transactional leadership was measured with a four-item index based on Yukl (1997). An example item is “My supervisor explains what has to be done in order to receive rewards such as a pay increase or promotion.” The response scale ranged from 1 (strongly disagree) to 5 (strongly agree). Procedural pay-setting justice was assessed with four of the seven items developed by Colquitt (2001), adjusted to capture perceptions of procedural justice in connection with pay setting. An example item is “To what extent has the pay-setting process been based on accurate information?” with the response options ranging from 1 (to a very small extent) to 5 (to a very large extent).

#### 3.2.2. Outcome variables

Among the work-related outcomes, task performance was measured with five items (e.g., “For the past 3 months, I have managed to plan my work so that it was done on time”) based on Koopmans et al. (2014). Turnover intention was measured with a single item (“I feel like resigning from my current employment”) derived from a multi-item measure (Sjöberg and Sverke, 2000). For both variables, the response scale ranged from 1 (strongly disagree) to 5 (strongly agree). Among the health-related outcomes, self-rated health was measured with one item, “How would you rate your general state of health?” (Odéen et al., 2013), with the response options (1) very poor, (2) rather poor, (3) neither good nor poor, (4) rather good, and (5) very good. Work-related exhaustion was assessed with a single item, “I feel completely exhausted when the work day is over,” from the Maslach Burnout Inventory—General survey (Maslach et al., 1996). The response scale for this item ranged from 1 (strongly disagree) to 5 (strongly agree).

#### 3.2.3. Demographic background variables

Data on age (years) and sex (1 = woman, 0 = man) were derived from national registers, while education level (1 = university, 0 = lower), managerial status (1 = yes, 0 = no), and employment status (1 = white-collar workers and academics, 0 = blue-collar workers) were provided by the participants themselves.

#### 3.2.4. Psychosocial work environment factors

For this block of variables, all items were self-assessed by the participants on a response scale that ranged from 1 (strongly disagree) to 5 (strongly agree). Employees’ job demands were captured by three items (e.g., “I fairly often have to work under heavy time pressure”) based on Beehr et al. (1976). Job control was captured using three items (e.g., “I can make my own decisions on how to organize my work”) drawn from various measures of job autonomy (Hackman and Oldham, 1975; Walsh et al., 1980; Sverke and Sjöberg, 1994). Finally, we used six items to measure social support that were based on measures of collegial (e.g., “There is always a co-worker to turn to when I encounter problems at work”) and managerial (e.g., “I always receive help from my manager when difficulties in my work arise”) support (Näswall et al., 2006).

### 3.3. Data analysis

#### 3.3.1. Identifying latent profiles

Latent profile analysis (LPA) was used to identify subpopulations characterized by various combinations of levels of compensation characteristics. LPA assumes that associations between statistical indicators can be explained by a categorical latent variable representing different combinations, that is, profiles. It is a type of mixture modeling (Muthén and Muthén, 1998-2017) that enabled us to cluster individuals into profiles comprising individuals with similar patterns of various circumstances and perceptions within a heterogeneous population.

In accordance with recommendations in previous studies (e.g., Nylund et al., 2007), the number of latent profiles (we tested 1–7 solutions) was determined by inspecting the Bayesian information criterion (BIC) and the sample-size adjusted BIC (SABIC), for which lower values indicate better fit. The enumeration process was also based on theoretical meaningfulness (Howard et al., 2016; Morin et al., 2018). The analysis was conducted using Mplus 8.3 (Muthén and Muthén, 1998-2017), using full information maximum likelihood estimation to handle missing data.^1^

As concerns BIC and SABIC, we also assembled elbow plots to show any gains in these fit indices after adding new solutions.^2^ The bootstrap likelihood ratio test (BLRT; Peel and McLachlan, 2000) was also utilized to compare solutions with different numbers of latent profiles, where a non-significant *p*-value indicates that a solution with k profiles fits the data better than a solution with k-1 profiles. We also relied on estimates about the proportion of individuals in each profile (where 5% is typically considered a minimum) and posterior probabilities (for which values greater than at least 0.70 indicate that the solution found can be interpreted; Nylund et al., 2007). We also considered the interpretability of the profiles by examining how the profiles differed in terms of the mean values for the variables that served as input to the LPA (i.e., the compensation characteristics). In addition, we calculated entropy (where values close to 1, typically above 0.70, indicate a better accuracy in the classification; Celeux and Soromenho, 1996). According to previous research, BIC and SABIC might be more reliable when entropy is high (~0.80), whereas BLRT may be a better indicator when entropy is very low (~0.50; Diallo et al., 2017). Thus, we took the level of entropy obtained into account before deciding whether to rely on BIC and SABIC or the BLRT. For transparency reasons, we also report the Lo–Mendell–Rubin adjusted Likelihood Ratio Test (LMR-LRT; Lo et al., 2001).

#### 3.3.2. Investigating differences between latent profiles

The Bolck, Croon, and Hagenaars (BCH) approach (Bolck et al., 2004) was used to examine whether the latent profiles differed with respect to outcome variables, demographic background variables, and psychosocial work environment factors, using Mplus. In the BCH procedure, analyses can be used without shifting the character of the profiles themselves while taking posterior probability levels of the profiles into account in the difference testing (for a review, see Asparouhov and Muthén, 2014). Wald Chi-square tests were used to test for differences between specific profiles.

## 4. Results

### 4.1. Identification of latent profiles

Table 2 presents the fit statistics for the seven profile solutions tested, based on the compensation characteristics, and shows the proportion of individuals assigned to each profile and the associated posterior probabilities. The BIC as well as the SABIC continued to improve (i.e., decrease) and the BLRT remained significant at the 0.001 level for all seven profile solutions. Entropy levels were relatively constant for the potential solutions involving three to seven profiles (ranging between 0.73 and 0.78), all slightly below 0.80, with solution 7 rendering the highest (best) entropy level at 0.78. The found entropy was closer to 0.80 than 0.50. Thus, BIC and SABIC guided the decision about the number of latent profiles. Both BIC and SABIC failed to reach a minimum.

**Table 2:** Fit indices for the latent profile models based on the compensation characteristics.

Solution	BIC	SABIC	BLRT	Proportion of total counts	Posterior probability	Entropy	LMR-LRT
1	10,849	10,824	–	[1.00]	[1.00]	[1.00]	–
2	10,490	10,449	394.21***	[0.49; 0.51]	[0.87; 0.88]	0.59	383.33***
3	10,386	10,328	139.74***	[0.14; 0.30; 0.56]	[0.87; 0.88; 0.88]	0.74	135.88***
4	10,302	10,229	118.46***	[0.12; 0.23; 0.23; 0.42]	[0.83; 0.85; 0.85; 0.85]	0.72	115.19***
5	10,217	10,128	120.08***	[0.11; 0.20; 0.23; 0.23; 0.23]	[0.81; 0.82; 0.83; 0.84; 0.85]	0.75	116.77*
**6**	**10,179**	**10,074**	73.36***	**[0.06; 0.09; 0.19; 0.20; 0.23; 0.23]**	**[0.74; 0.81; 0.82; 0.83; 0.86; 0.86]**	**0.75**	71.34***
7	10,135	10,014	79.31***	[0.06; 0.07; 0.08; 0.18; 0.20; 0.20; 0.21]	[0.80; 0.82; 0.82; 0.83; 0.85; 0.85; 0.85]	0.77	77.11

*N* = 1,146. *p < 0.05, **p < 0.01, ***p < 0.001. – Not applicable. BIC, Bayesian information criterion; SABIC, sample-size adjusted; LMR-LRT, Lo–Mendell–Rubin adjusted likelihood ratio test; BLRT, bootstrapped log-likelihood ratio test. Bold indicates best-fitting model.

**Figure 1:**
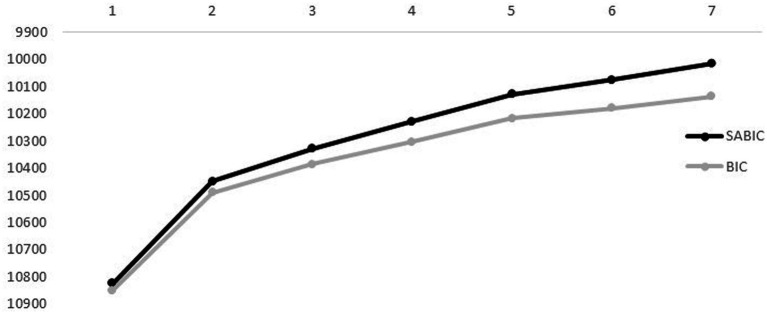
Elbow plot for the Bayesian information criterion (BIC) and the sample-size adjusted Bayesian information criterion (SABIC) for the seven tested profile solutions.

The elbow plots for BIC and SABIC, presented in Figure 1, show that when the six-profile solution was added, the decrease in BIC and SABIC was somewhat smaller. Thus, we compared the fifth solution (after which the slope flattened) to the solution with one fewer profile (i.e., solution 4) and the solution with one more profile (i.e., solution 6). However, as the statistical indicators indicated that the solution with seven profiles further decreased the BIC and SABIC while BLRT continued to remain significant, the theoretical meaningfulness of the seventh solution was also investigated.

It was clear that the fifth solution added more theoretical meaningfulness than the fourth solution did. Existing profiles became more clearly separated from each other in terms of mean values in the input variables, and a fifth profile emerged that was of a completely different character than the first four. In the sixth solution, a profile with low levels of perceived horizontal pay dispersion emerged, and this profile was clearly different to other profiles with low levels of horizontal pay dispersion. The average salary of individuals in this profile was also very close to that of another profile which had high horizontal pay dispersion. Thus, the sixth solution might shed light on how those groups with high or low pay dispersions and with relatively low pay relate to work-related and health-related outcomes. Adding a sixth solution also resulted in one profile having clearly low transactional leadership along with high procedural pay-setting justice, a composition which may allow comparisons with other combinations of these variables (e.g., high/high or low/low).

The theoretical value of adding a seventh profile was smaller. The seventh solution seemed to split the profile with the very highest incomes into two profiles, with those with generally higher mean values in one of the profiles, and those with slightly more moderate mean values in the other. Thus, compared to the six-profile solution, the seventh profile added relatively little in terms of theoretical meaningfulness. A disadvantage with choosing the six-profile solution was that the posterior probabilities were slightly lower as compared to the five-profile solution. All six profiles included more than the recommended 5 percent minimum proportion of the sample. Based on this, the solution containing six profiles was chosen.

In terms of interpretations of the mean levels of the compensation characteristics, all profiles contained unique patterns. Based on these mean levels, profiles were first categorized based on their relative levels of pay (low pay, slightly below average pay, average pay, and high pay) in line with the national average of pay levels for private sector employees in Sweden in 2016. Labels were then given based on the level of perceived horizontal pay dispersion (compressed, moderately dispersed, highly dispersed) and procedural quality (i.e., referring to low, moderate, high, or mixed mean levels for transactional leadership and procedural pay-setting justice). Table 3 presents the mean levels of the compensation characteristics for the six latent profiles. The profiles were labeled as follows:

**Table 3:** Mean levels of the input variables for the six latent profiles.

Profile	Low pay	Slightly below average pay		Average pay		High pay	Total
	1	2	3	4	5	6	
	*Compressed with low procedural quality*	*Compressed with mixed levels of procedural quality*	*Highly dispersed with low procedural quality*	*Compressed with high procedural quality*	*Moderately dispersed with moderate procedural quality*	*Highly dispersed with high procedural quality*	
Monthly pay level (log)	10.18	10.30	10.30	10.39	10.38	10.53	10.37
Perceived horizontal pay dispersion	1.34	1.43	4.61	1.31	3.12	3.86	3.03
Transactional leadership	1.83	2.46	2.06	4.02	2.64	3.70	2.81
Procedural pay-setting justice	1.71	3.47	2.35	4.26	3.05	3.99	3.23
*N* (%)	69 (6)	220 (19)	228 (20)	101 (9)	262 (23)	266 (23)	1,146 (100)

#### 4.1.1. Profile 1. Low pay: Compressed with low procedural quality

This profile was characterized by low mean levels for all compensation characteristics. It contained about 6% of the employees in the sample.

#### 4.1.2. Profile 2. Slightly below average pay: Compressed with mixed procedural quality

The average pay level in this profile, which contained about 19% of the sample, was quite low. It was also characterized by low levels of perceived horizontal pay dispersion. In regard to procedural quality, the levels of transactional leadership and procedural pay-setting justice were mixed, with the former being relatively low and the latter being relatively high.

#### 4.1.3. Profile 3. Slightly below average pay: Highly dispersed with low procedural quality

This profile, representing about 20% of the sample, was characterized by low levels for all compensation characteristics except perceived horizontal pay dispersion, which was at a high level.

#### 4.1.4. Profile 4. Average pay: Compressed with high procedural quality

The levels for all compensation characteristics were high in this profile, except for pay level, which was close to the national average, and perceived horizontal pay dispersion, which was low. About 9% of the employees in the sample were in this profile.

#### 4.1.5. Profile 5. Average pay: Moderately dispersed with moderate procedural quality

This profile (about 23% of the sample) was characterized by moderate values for all compensation characteristics.

#### 4.1.6. Profile 6. High pay: Highly dispersed with high procedural quality

This profile, comprising about 23% of the sample, was characterized by high values for the compensation characteristics.

### 4.2. Differences between latent profiles

Table 4 presents the mean levels for the work-related and health-related outcomes for the six latent profiles. Table 5 shows the proportions (or means) for the demographic background variables, and the mean levels for the psychosocial work environment factors. The tables also show the results of the Wald Chi-square tests for differences between profiles.

**Table 4:** Wald *χ*^2^difference tests between the six latent profiles for the work-related and health-related outcomes.

Profile	Low pay	Slightly below average pay		Average pay		High pay	Total	Significant mean differences between profiles
	1	2	3	4	5	6		
	*Compressed with low procedural quality*	*Compressed with mixed levels of procedural quality*	*Highly dispersed with low procedural quality*	*Compressed with high procedural quality*	*Moderately dispersed with moderate procedural quality*	*Highly dispersed with high procedural quality*		
Task performance	3.91	3.88	3.95	4.37	3.65	4.07	3.92	4 > 1–3&5–6; 6 > 1–2&5; 2–3 > 5
Turnover intention	2.14	1.62	2.37	1.22	1.99	1.54	1.83	4 < 1–3&5–6; 2&6 < 1&3&5; 5 < 3
Self-rated health	3.82	4.02	3.91	4.31	3.99	4.30	4.08	4&6 > 1–3&5
Work-related exhaustion	3.59	2.99	3.69	2.79	3.25	2.90	3.19	4&6 < 1&3&5; 2 < 1&3; 5 < 3
*N* (%)	69 (6)	220 (19)	228 (20)	101 (9)	262 (23)	266 (23)	1,146 (100)	

Significant differences between profiles based on Wald *χ*^2^ below *p* < 0.05.

**Table 5:** Wald *χ*^2^ difference tests between the six latent profiles for the demographic background variables and psychosocial work environment factors.

Profile	Low pay	Slightly below average pay		Average pay		High pay	Total	Significant mean differences between profiles
	1	2	3	4	5	6		
	*Compressed with low procedural quality*	*Compressed with mixed levels of procedural quality*	*Highly dispersed with low procedural quality*	*Compressed with high procedural quality*	*Moderately dispersed with moderate procedural quality*	*Highly dispersed with high procedural quality*		
**Demographic background variables**								
Age (years)	51	47	44	46	48	46	47	1&5 > 3&6; 1 > 4; 2 > 3
Education (university degree)	26	39	45	42	47	52	45	3&5–6 > 1; 6 > 2
Sex (woman)	33	18	46	31	27	21	29	3 > 2&4–6
Managerial status (manager)	10	8	12	42	16	37	21	4&6 > 1–3&5
Employment status (white-collar workers)	23	26	44	62	60	80	54	1–2 < 3–6; 3 < 4–6; 6 > 4–5
**Psychosocial work environment factors**								
Job demands	3.14	3.26	3.73	2.94	3.43	3.24	3.35	4 < 3&5; 3 > 1–2&6
Job control	2.71	3.62	3.28	4.55	3.65	4.28	3.74	4 > 1–3&5–6; 6 > 1–3&5; 2&5 > 1&3; 3 > 1
Social support	2.59	3.45	3.02	4.43	3.29	4.08	3.51	4 > 1–3&5–6; 6 > 1–3&5; 2&5 > 1&3; 3 > 1
*N* (%)	69 (6)	220 (19)	228 (20)	101 (9)	262 (23)	266 (23)	1,146 (100)	

Significant differences between profiles based on Wald *χ*^2^ below *p* < 0.05.

#### 4.2.1. Profile 1. Low pay: Compressed with low procedural quality

In terms of outcomes, Profile 1 had around average levels of task performance, higher levels of turnover intention, and higher levels of work-related exhaustion and slightly lower levels of self-rated health as compared with the sample average. Employees in this profile were slightly older on average (although the differences regarding age were quite marginal between profiles), an overwhelming majority did not have a university education, and around one-third were woman. Few were managers, and a clear majority worked in blue-collar occupations. This profile was also characterized by relatively low levels for the psychosocial work environment factors, except for job demands, which was moderate.

#### 4.2.2. Profile 2. Slightly below average pay: Compressed with mixed procedural quality

Regarding the outcome variables, this profile was characterized by average levels of task performance and self-rated health and relatively low levels of turnover intentions and work-related exhaustion. This profile was average in terms of age but was characterized by a lower level of education and a lower proportion of women as compared to several other profiles (around one-fifth were women). In addition, the proportions of managers and white-collar workers were substantially lower than the sample average. In terms of psychosocial work environment factors, this profile was characterized by around average values of demands, control, and social support.

#### 4.2.3. Profile 3. Slightly below average pay: Highly dispersed with low procedural quality

The levels of task performance and self-rated health were around average, whereas individuals in this profile reported the highest levels of turnover intention and work-related exhaustion. This profile was around average in terms of age and education and had a higher proportion of women as compared to several other profiles. It was also characterized by below-average levels in terms of the proportions of managers and white-collar workers. Employees in this profile had high job demands and low levels of job control and social support.

#### 4.2.4. Profile 4. Average pay: Compressed with high procedural quality

In terms of the outcomes, this profile was characterized by the highest task performance and the lowest level of turnover intention as compared to the other profiles. It was also characterized by high levels of self-rated health, and low levels of work-related exhaustion. The profile was quite average with regard to age, education, and sex, while the proportion of managers was high, and the proportion of white-collar workers was slightly higher than the sample mean. This profile was also characterized by a low level of perceived job demands as well as by high levels of perceived job control and social support.

#### 4.2.5. Profile 5. Average pay: Moderately dispersed with moderate procedural quality

Regarding work-related and health-related outcomes for this profile, the level of task performance was below average, whereas turnover intention and work-related exhaustion were slightly higher than the average, and self-rated health was average, as compared to the total sample. This profile was quite average in terms of age, containing a slightly higher proportion of individuals with university education, and somewhat more women, as compared to some other profiles. It contained a below-average proportion of managers, but a slightly above-average percentage of white-collar employees. The profile was also characterized by moderate job demands and job control, while the level of social support was below average.

#### 4.2.6. Profile 6. High pay: Highly dispersed with high procedural quality

Concerning the outcome variables, this profile was characterized by higher levels of task performance and self-rated health, and by lower levels of turnover intention and exhaustion, as compared to most other profiles. This profile was quite average in regard to age (although employees were slightly younger than employees in a few other profiles), a slight majority had a university education, and around one-fifth were women. There was also an overrepresentation of employees with managerial positions, and an overwhelming majority worked in white-collar occupations. It was also characterized by high job control and high social support levels and by average levels of job demands.

## 5. Discussion

While there are contrasting research perspectives (e.g., expectancy theory vs. SDT) on how pay systems should be designed to create the best possible outcomes for organizations (e.g., improved performance) and their employees (e.g., maintained health; see, e.g., Gagné and Forest, 2008; Shaw and Gupta, 2015; Lazear, 2018; Gagné and Forest, 2020), relatively little attention has been given to examining shared perceptions of relevant pay-related factors (i.e., a person-centered approach) in the labor market. Given this, our first aim was to identify different latent profiles of individuals based on compensation characteristics (regarding pay level, perceived horizontal pay dispersion, transactional leadership, and procedural pay-setting justice) using a nationally representative sample of private sector employees in Sweden. Our second aim was to investigate differences between these profiles in work-related (task performance and turnover intention) and health-related outcomes (self-rated health and work-related exhaustion). To further elaborate on the meaningfulness of the revealed differences in the outcomes, the third aim was to investigate differences between the profiles in terms of demographic background variables (age, education level, sex, managerial status, and employment status) and psychosocial work environment factors (job demands, job control, and social support).

### 5.1. Compensation profiles

The latent profile analysis identified six compensation profiles. On average, the first three profiles were characterized by pay levels either lower than (Profile 1) or slightly below the national average (profiles 2 and 3). The latter three profiles had either average (profiles 4 and 5) or high (Profile 6) pay levels. Among the profiles with pay levels below the national average, two were characterized by low levels of perceived horizontal pay dispersion (profiles 1 and 2), levels considered by previous research to reflect pay compression (Bloom, 1999), and one by high levels (Profile 3). The profiles with average to high pay levels also differed regarding perceived horizontal pay dispersion: one was characterized by low levels (Profile 4), another by moderate levels (Profile 5), and the last by high levels (Profile 6). Further increasing the differences between the profiles were the levels of transactional leadership [included to reflect pay-related instrumentality provided by pay-setting managers, as is emphasized by certain theoretical perspectives (inspired by expectancy theory; Vroom, 1964)] and the levels of procedural pay-setting justice (included to reflect the fairness of pay setting and inspired by justice theories; e.g., Leventhal, 1980; Colquitt, 2001; see Stråberg, 2010). The means levels for procedural quality differed substantially for only one profile (Profile 2), where transactional leadership was relatively low, while procedural pay-setting justice was relatively high (thus this pattern was labeled “mixed”). As the levels for these two variables were similar across most of the profiles, they were together considered to reflect procedural (pay-setting) quality, based on assumptions in pay-related theoretical perspectives (e.g., Shaw, 2014; Han et al., 2015). Two profiles (Profile 1 and Profile 3) were characterized by low procedural quality. Employees in another profile (Profile 5) perceived moderate procedural quality while those in two other profiles (4 and 6) perceived high procedural quality.

The character of the six detected latent (compensation) profiles reflects an enlarged picture of how the integrative nature of compensation characteristics (with particular attention being put on low and high values) makes for differences in work-related and health-related outcomes. Arguably, applying latent profile analysis (LPA; Gibson, 1959) to research of compensation characteristics is compatible with many theoretical perspectives (e.g., Gagné and Forest, 2008; Shaw, 2014) which claim that—but rather seldom explore if (because they use variable-centered approaches)—employees’ overall perceptions of certain compensation characteristics are crucial to understanding how compensation systems may improve or worsen work-related and health-related outcomes.

### 5.2. Key findings regarding work-related and health-related outcomes

A first key finding is that the two profiles with the most favorable levels in the work-related and health-related outcomes were profiles 4 and 6. These profiles had the highest levels of self-rated health and the lowest levels of work-related exhaustion. They were also characterized by better task performance and lower turnover intention, although Profile 4 (Average pay, Compressed with high procedural quality) had slightly higher task performance and lower turnover intention as compared to Profile 6 (High pay, Highly dispersed with high procedural quality). These two profiles were characterized by average to high pay combined with high procedural quality (in terms of procedural pay-setting justice and transactional leadership). This finding is in line with previous income-related research (Ettner, 1996; Kuvaas, 2006; Kuvaas et al., 2016; Thibault-Landry et al., 2017; Jebb et al., 2018), which suggests that higher income is associated with more positive work-related attitudes and better health. It is also in line with compensation-related perspectives with their roots in justice theories (e.g., Leventhal, 1980) and equity theory (Adams, 1965), suggesting that procedural quality may determine to what extent pay systems result in positive work-related and health-related outcomes, even in cases of differing degrees of pay dispersion (Gagné et al., 2023). In contrast, the combination of low pay and unfair procedures was associated with higher turnover intention, poorer self-rated health, and higher work-related exhaustion, irrespective of pay compression (Profile 1) or high pay dispersion (Profile 3). It thus seems that a decent pay (average or high) perceived as fairly set is very important.

A second key finding concerns the role of pay dispersion. Comparing the two profiles with average pay indicates that Profile 4 (Compressed with high procedural quality) was characterized by higher levels of task performance and self-rated health as well as lower levels of turnover intention and work-related exhaustion than Profile 5 (Moderately dispersed with moderate procedural quality). This finding indicates that pay compression may be associated with beneficial outcomes, especially when combined with high procedural pay-setting justice and transactional leadership. Such an interpretation is in line with previous research highlighting that high horizontal pay dispersion is negatively related to job performance (e.g., Bloom, 1999). This finding indicates a certain advantage of pay compression, which is in line with some of the previous research (Bloom and Michel, 2002), but also suggests that such an outcome is contingent on the pay procedures being perceived as fair by the employees (Olafsen et al., 2015). In conclusion, both compression and fairness seem important for work-related and health-related outcomes.

A third key finding concerns the role of procedural quality (in terms of procedural pay-setting justice and transactional leadership). In general, the tendency was that profiles characterized by higher levels of procedural quality had more favorable outcomes than those with lower levels of procedural quality. This is in line with previous research on organizational (Colquitt, 2001) and pay-setting justice (Stråberg, 2010) as well as transactional leadership (Yukl, 1999; Judge and Piccolo, 2004; Rowold and Schlotz, 2009). However, this general tendency was also qualified by other factors describing the profiles. As an example, profiles 4 and 6, which both had high procedural quality, had the most favorable health outcomes, but Profile 4 (average pay and pay compression) had slightly higher job performance and slightly lower turnover intention as compared to Profile 6 (high pay and high pay dispersion). This could indicate that pay compression may have beneficial consequences (e.g., Bucciol et al., 2014), at least if combined with high procedural quality (Shaw, 2014). For instance, the highest levels of exhaustion were found for Profile 1 (Low pay, compressed with low procedural quality) and Profile 3 (Slightly below average pay, highly dispersed with low procedural quality), whereas the lowest levels were observed for Profile 4 (Average pay, compressed with high procedural quality) and Profile 6 (High pay, highly dispersed with high procedural quality). In addition, Profile 2 (Slightly below average pay, Compressed with mixed levels of procedural quality) also had lower exhaustion as compared to profiles 1 and 3, although it is difficult to determine if this depends on the degree of pay compression or procedural quality or even pay level. These findings indicate that pay level (*cf.* Ettner, 1996), the degree of pay dispersion (*cf.* Dahl and Pierce, 2020), and procedural quality (*cf.* Shaw, 2014) may all be relevant for health outcomes such as work-related exhaustion. Reflecting this, an interesting difference was between profiles 3 and 4, where the lower levels of exhaustion characterizing Profile 4 could be attributed to the finding that individuals in this profile had slightly higher pay and substantially better procedural quality as compared to Profile 3, but also that Profile 4 was characterized by pay compression, whereas Profile 3 was highly dispersed. Another interesting difference illustrating this concerned the two profiles with slightly below average pay, where Profile 3 (highly dispersed with low procedural quality) had higher work-related exhaustion (and higher turnover intention) than Profile 2 (compressed with mixed levels of procedural quality).

These key findings indicate that pay level, perceptions of horizontal pay dispersion as well as procedural quality (procedural pay-setting justice and transactional leadership) may all matter for work-related and health-related outcomes. However, it is important to consider their combinations, rather than to study them separately, to understand their effects on employee outcomes.

### 5.3. Characterizations based on demographic and psychosocial work environment factors

Based on the data on demographic background factors (age, education level, sex, managerial status, and employment status) and psychosocial work environment factors (job demands, job control, and social support), two main pools of profiles were identified that showed rather clear similarities internally (i.e., with other profiles in their pool) and differences externally (i.e., with other profiles outside of their pool). The first pool, comprising profiles 4 (Average pay: Compressed with high procedural quality) and 6 (High pay: Highly dispersed with high procedural quality), showed many similarities regarding their proportions (or means) for the demographic background variables, albeit with some exceptions (the main exception was that Profile 6 had a higher representation of white-collar workers). In regard to the psychosocial factors, which were included based on the Demand–Control–Support model (Karasek and Theorell, 1990), low levels of job demands and high levels of job control and social support reflect better work environments than do other combinations of these variables. Both profiles in the first pool were characterized by better psychosocial work environment levels as compared to the other profiles (internally, however, Profile 4 had higher levels of job control and social support compared to Profile 6).

Among the remaining profiles (1–3 and 5), they too showed more similarities in terms of demographic and psychosocial characteristics with one another than with the two other profiles (i.e., profiles 4 and 6), thus they formed a second pool of profiles. However, there were more important differences among the profiles in the second pool, as well as some tendencies similar to those found in the first pool. Regarding demographic background variables, Profile 3 (Slightly below average pay: Highly dispersed with low procedural quality) and Profile 5 (Average pay: Moderately dispersed with moderate procedural quality) had larger proportions of university-educated employees and employees in white-collar occupations, as compared to profiles 1 and 2. In addition, Profile 5 showed some similar tendencies to those (e.g., regarding the proportion of white-collar workers) in the first pool of profiles (i.e., profiles 4 and 6). However, concerning psychosocial work environment factors, Profile 2 (Slightly below average pay: Compressed with mixed procedural quality) was characterized by better psychosocial work environment levels than some of the others (mainly profiles 1 and 3). Another important difference concerned the profile with the lowest average pay, Profile 1 (Low pay: Compressed with low procedural quality), which to some extent stood out on its own. Regarding demographics, Profile 1 contained the lowest proportion of university-educated employees and the largest proportion of blue-collar workers. It was also characterized by worse psychosocial work environment levels (although its job demands levels were slightly lower than Profile 3’s). Relatedly, previous research has highlighted that poor psychosocial work environments are associated with a large number of negative work-related and health-related outcomes (e.g., Schaufeli and Taris, 2014).

Thus, our findings concerning characterizations of the profiles demonstrate that their differences in work-related and health-related outcomes were likely impacted by their general demographic and psychosocial characteristics. Overall, however, it was identified that certain profiles in the first pool (4 and 6) and in the second (1–3 and 5) pool were quite comparable with other profiles in their respective pools, but not very much with others—albeit with some exceptions (mainly that Profile 5 showed similarities also with the profiles outside of its respective pool, and that Profile 1’s demographic and psychosocial character stood out partly on its own). This indicates that the effects of employee pay and pay setting (i.e., compensation characteristics) may depend on both psychosocial and demographic factors, thus suggesting that such aspects need to be considered in future research to increase our understanding of how compensation characteristics associate with various outcomes.

### 5.4. Methodological considerations

Although this study, based on the use of profiles of individuals with different combinations of pay-related factors, provides useful insights about the importance of considering combinations of pay characteristics in determining employee outcomes, there are a number of potential methodological limitations in this study. First, the cross-sectional design does not allow us to conclude that the compensation profiles gave rise to the work-related and health-related outcomes (Shadish et al., 2001). While our assumptions of directionality (i.e., that compensation characteristics drive work-related and health-related outcomes rather than the reverse) are consistent with previous theory (e.g., Ganster et al., 2011; Shaw, 2014) and research findings (Cerasoli et al., 2014; Dahl and Pierce, 2020), the possibility of reverse causation cannot be ruled out. The present findings thus await replication by other research that can shed more light on how certain combinations of compensation characteristics relate to work-related and health-related outcomes.

A second limitation, which applies to most survey research, is that we relied on self-reported measures (for all variables except some demographics), which may result in common method variance (CMV) and inflated associations (Frese and Zapf, 1999). However, it has been argued that the risk of CMV is often overstated (Spector, 2006) and that self-reports are particularly suitable for studying individuals’ perceptions of characteristics at work and their reactions (*cf.* Conway and Lance, 2010) because of the subjective nature of such experiences. Yet, future research may wish to replicate the present findings by using other sources for outcome measures (e.g., supervisor assessments of performance and register-based indicators of job performance, turnover, and health).

Despite using a nationally representative sample of private sector employees that provided variability in pay characteristics, a third limitation concerns the generalizability of our findings. While we used a nationally representative sample of private sector employees, there may be distinguishing features of the private sector that limit generalizability to public sector employees. In addition, the data were collected in only one country (Sweden), suggesting that the present findings need replication in countries with other salient characteristics concerning pay setting.

Finally, it should be noted that the identification of profiles was based on specific indicators aligning to form different groups characterized by different patterns of compensation characteristics. While we have made the case for the relevance of pay level, pay dispersion, and perceptions of transactional leadership and procedural justice, there are other salient characteristics and perceptions of the pay-setting process that may form a different configuration of profiles. For instance, in future research, it may be relevant to include measures of how strong the link between performance and monetary rewards is (Deci et al., 1999; Gagné and Deci, 2005; Cerasoli et al., 2014) to more reliably determine the significance of pay-related instrumentality (beyond transactional leadership) for employee work attitudes, work-related behavior, and health.

### 5.5. Future research directions

While our study focused on compensation experiences and perceptions in the Swedish private sector, it is unclear from our study whether the average proportions of various pay system types (e.g., performance-based pay systems as well as traditional pay systems, where employee pay is based on employment-related factors such as seniority, or egalitarian pay systems, where pay is solely based on the work role; Pfeffer, 1997, 1998; Bloom, 1999; Maaniemi, 2013) among the employees in the profiles may have contributed to the differences in work-related and health-related outcomes found between the profiles.^3^ It is thus recommended that future research include pay-system type as a profile predictor. Then, autonomous (meaning- or engagement-based motivation) and extrinsically controlled work motivation (personal gain-based motivation) may also be included as potential mediators of the associations between compensation profiles and outcomes. These types of motivation may be important to include given that there are differing theoretical views on how compensation experiences relate to motivation [see Shaw and Gupta (2015) and Gagné and Deci (2005) for contrasting perspectives]. On the one hand, assumptions based on expectancy theory (Vroom, 1964) assert that performance-based pay systems have an advantage over traditional and egalitarian pay systems in that they drive employee motivation and encourage retention among top performers (Shaw, 2014, 2015). On the other hand, SDT (Deci and Ryan, 1985) assumes that performance-based pay systems encourage extrinsically controlled work motivation, which the theory predicts may contribute negatively to work-related and health-related outcomes (e.g., Gagné and Deci, 2005; Gagné and Forest, 2020).

## 6. Conclusion

By investigating similarities among employees with regard to various compensation characteristics (regarding pay levels, and perceptions of horizontal pay dispersion, transactional leadership, and procedural pay-setting justice) through latent profile analysis (Gibson, 1959), our study contributes with a new take on the study of how pay-system experiences and perceptions may impact work-related and health-related outcomes. Based on data from a nationally representative sample of private sector employees in Sweden, combining various characteristics of pay setting allowed us to identify six distinct profiles. On the whole, three key findings emerged. First, higher levels of pay were generally associated with more positive work-related and health-related outcomes, especially when combined with perceptions of good procedural quality (procedural pay-setting justice and transactional leadership). Second, in terms of perceived horizontal pay dispersion, our findings indicate that pay compression under certain circumstances—especially good procedural quality—may be associated with beneficial outcomes. Third, procedural quality was generally associated with better performance, lower turnover intention, better self-rated health, and lower work-related exhaustion, although such positive effects may be contingent upon pay level and perceived horizontal pay dispersion. Taken together, these key findings suggest that pay level, perceptions of horizontal pay dispersion, and procedural quality (procedural pay-setting justice and transactional leadership) may all be important for work-related and health-related outcomes, but that it is crucial to consider their combinations to understand how compensation characteristics may affect employee outcomes.

However, these key findings should be considered in light of the demographic and psychosocial factors characterizing the profiles. In this respect, a main finding was that the two profiles showing the most positive outcomes contrasted strongly with other profiles with more negative outcomes (e.g., they had greater managerial representation and a better psychosocial work environment), thus suggesting that their more favorable outcomes may partly be explained by demographic and psychosocial factors.

This is, to the best of our knowledge, the first study to apply a person-oriented approach to pay setting. Rather than to study direct associations between various compensation characteristics and outcomes, we identified groups of individuals (latent profiles) with different combinations of pay level and perceptions of horizontal pay dispersion, transactional leadership, and procedural pay-setting justice—and compared these profiles in terms of task performance, turnover intention, general health, and work-related exhaustion.

A practical implication of the present results is that pay setting needs to consider the *combination* of several compensation characteristics. More specifically, combinations of a decent pay level (average or high) and high procedural quality (in terms of procedural pay-setting justice and transactional leadership) thus appear to stimulate positive work-related and health-related outcomes. In terms of perceived horizontal pay dispersion, pay compression may have favorable consequences, especially if combined with high procedural quality. This means that organizations should place effort on the quality of the pay setting—especially the justice aspect—by having clear pay criteria, being transparent about the procedures used to evaluate employee performance and to set pay, and providing employees opportunities for participation in the pay-setting process. Our results show that employees who perceive the pay setting as just and understand why they get a certain pay also report higher task performance, lower turnover intention, better general health, and less work-related exhaustion. In conclusion, while pay level, perceptions of horizontal pay dispersion, and procedural quality may all matter for employee outcomes, it is important to consider their combinations in order to stimulate job performance, retain employees, and maintaining good health among the staff. This may be especially relevant in a post-pandemic period, when many organizations have undergone change and need to attract, motivate, and retain employees.

## Data availability statement

The datasets presented in this article are not readily available because our data cannot be publicly shared. Requests to access the datasets should be directed to alexander.nordgren@psychology.su.se.

## Ethics statement

The studies involving human participants were reviewed and approved by The Regional Ethics Committee in Stockholm (ref. no. 2015/1733-31/5). The participants provided their written informed consent to participate in this study.

## Author contributions

AN provided the idea for the study, carried out the statistical analyses, in discussion with MG, JH, HF, and MS, and wrote the first draft of the paper. AN, MG, JH, HF, and MS participated in the conceptualization as well as the design and analytical approach of the study. MG, JH, HF, and MS contributed to revisions and writing of the final paper. All authors contributed to the article and approved the submitted version.

## Funding

This study was financed by a grant to MS from the Confederation of Swedish Enterprise (grant no. 313002).

## Conflict of interest

The authors declare that the research was conducted in the absence of any commercial or financial relationships that could be construed as a potential conflict of interest.

## Publisher’s note

All claims expressed in this article are solely those of the authors and do not necessarily represent those of their affiliated organizations, or those of the publisher, the editors and the reviewers. Any product that may be evaluated in this article, or claim that may be made by its manufacturer, is not guaranteed or endorsed by the publisher.

## Footnotes

^1^The number of random starts was pre-set to maximum 5,000. Obtaining no more solutions after 5,000 starts may indicate that a maximum number of solutions has been reached. Furthermore, replication using log-values for all converging solutions was carried out. This was done in line with recommendations for latent class/profile analysis made by the developers of the statistical program in their MPLUS User’s guide (Muthén and Muthén, 1998-2017), and on their website chat forum: http://www.statmodel.com/discussion.

^2^According to recommendations in previous research, elbow plots can be used in the enumeration process (Morin and Marsh, 2015), where a point where the slope flattens indicates that theoretical saturation may have occurred or be near. The theoretical meaningfulness of this solution can then be compared to that of one lower, and one higher solution (and further solutions with even more profiles if BIC and SABIC continue to decrease). Among these solutions, the one rendering most theoretical meaningfulness should be chosen.

^3^In Sweden, those in occupations that typically demand a university degree, which is the case for most white-collar jobs and managerial positions, to a rather large extent work under performance-based pay systems. They are also common in occupations where women are overrepresented historically, even among blue-collar occupations. They are not common in blue-collar occupations where men are overrepresented historically. Notably, however, performance-based pay systems are generally less common among private sector employees than among public sector employees in Sweden (Hellgren et al., 2017; Swedish National Mediation Office, 2017).
